# Immune hub genes and a proof-of-concept prognostic signature in EBV-associated gastric carcinoma

**DOI:** 10.1016/j.isci.2026.115243

**Published:** 2026-03-05

**Authors:** Rui-zhen Huo, Ri-hong Yang, Ri-hua Zeng, Zi-cen Fang, Bin Li, Yu-hang Pan, Jin-rui Guo, Yan-xian Guan, Chun-kui Shao, Dan-ni Yu, Si-hong Liang, Yi-ting Shao, Yu Du, Jian-ning Chen

**Affiliations:** 1 Department of Pathology, The Third Affiliated Hospital, Sun Yat-sen University, Guangzhou 510630, China; 2 Department of Stomatology, The Third Affiliated Hospital, Sun Yat-sen University, Guangzhou 510630, China; 3 NMPA Key Laboratory for Research and Evaluation of Drug Metabolism & Guangdong Provincial Key Laboratory of New Drug Screening, School of Pharmaceutical Sciences, Southern Medical University, Guangzhou 510515, China

**Keywords:** Health sciences, Medicine, Medical specialty, Health informatics, Internal medicine, Oncology

## Abstract

Epstein-Barr virus-associated gastric carcinoma (EBVaGC) represents a distinct molecular subtype with unique immune characteristics but lacks subtype-specific prognostic tools for clinical management. We developed an immune-based prognostic signature by integrating transcriptomic profiling, immunohistochemical validation, and functional assays. PTPRC (CD45) and ITGB2 (CD18) were identified as central immune regulators with strong correlation (r = 0.78, *p* < 0.001) and significant enrichment in EBVaGC (CD45^+^ and CD18^+^: 6907 vs. 2876 and 542 vs. 160 cells/mm^2^, both *p* < 0.0001), co-localizing with CD68^+^ macrophages. The four-gene Immunoscore demonstrated robust risk stratification (C-index = 0.922; hazard ratio = 24.6, *p* = 0.002) and maintained discrimination in independent validation (C-index = 0.765; hazard ratio = 8.68, *p* = 0.0023). Functional assays confirmed CD18’s role in monocyte migration (62.6% reduction upon blockade, *p* < 0.001). This exploratory study establishes an immune-based prognostic model for EBVaGC, with potential to guide risk-adapted surveillance and immunotherapy selection pending multi-center validation.

## Highlights

• PTPRC (CD45) and ITGB2 (CD18) were identified as key immune regulators in EBVaGC
• A four-gene Immunoscore was developed and showed robust risk stratification in EBVaGC
• CD18 played an important role in monocyte migration and immune cell recruitment
• A prognostic model was established to guide risk-adapted surveillance and immunotherapy

## Graphical abstract

**Figure:**
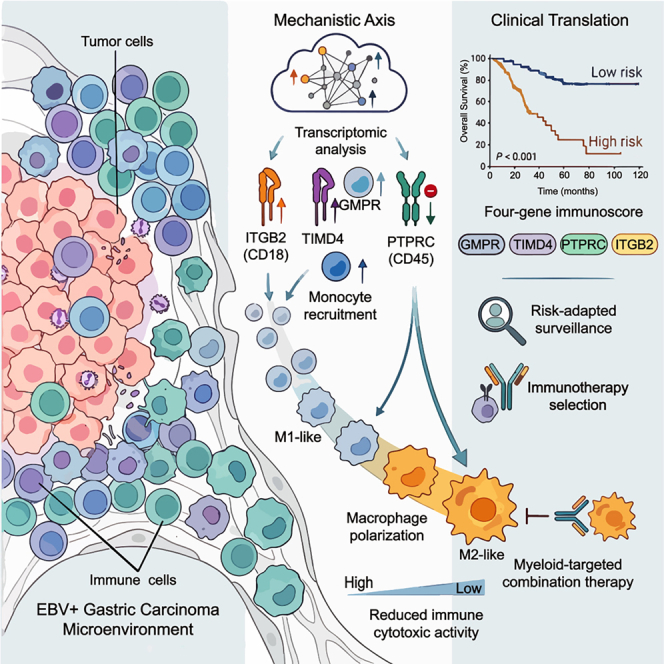

## Introduction

Epstein-Barr virus (EBV), the first identified oncogenic herpesvirus, is associated with several malignancies, including Burkitt lymphoma, Hodgkin lymphoma, nasopharyngeal carcinoma, and gastric carcinoma (GC).^1^ EBV-associated gastric carcinoma (EBVaGC) represents a distinct subtype with monoclonal viral integration, suggesting EBV as an early oncogenic driver.^2^

Accounting for approximately 10% of GC, EBVaGC predominantly affects males, arises in the proximal stomach, and generally portends a favorable prognosis with low nodal involvement.^3^^,^^4^^,^^5^ However, this favorable overall prognosis masks substantial intra-subtype heterogeneity: a clinically significant proportion of patients with EBVaGC still experience disease progression, recurrence, or cancer-related mortality. Conventional clinicopathological parameters fail to reliably identify these high-risk individuals.

EBVaGC features an immune-enriched microenvironment with extensive lymphocyte infiltration, high PD-L1 expression, and interferon pathway activation.^6^^,^^7^ However, immune evasion mechanisms—such as T cell exhaustion and M2 macrophage infiltration—contribute to a dichotomous immune landscape characterized by concurrent activation and suppression.^8^^,^^9^^,^^10^ This duality necessitates integrated immunogenomic approaches to capture clinically relevant immune features.

This unmet clinical need is compounded by the absence of validated EBVaGC-specific prognostic biomarkers. Despite these recognized molecular and immunological distinctions, clinical practice continues to apply standard GC protocols without subtype-tailored risk assessment. This proof-of-concept study aims to address the translational gap between the recognized distinctiveness of EBVaGC and available clinical management tools, establishing biological feasibility for precision biomarker development before large-scale validation efforts.

To address these knowledge gaps, we conducted an integrative transcriptomic study with the following objectives: (1) identify key immune regulatory genes specific to EBVaGC; (2) validate their expression and functional associations across independent datasets; (3) explore their potential as prognostic biomarkers through proof-of-concept modeling; and (4) provide a foundation for future large-scale validation studies.

## Results

### Identification of differentially expressed genes

**Figure 1:**
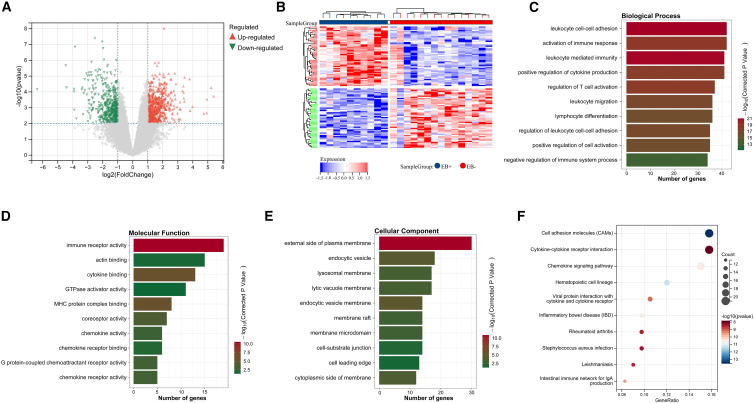
Discovery and functional enrichment overview (A) Volcano plot of differentially expressed genes (DEGs) between EBVaGC and EBVnGC in GSE51575. (B) Heatmap of top-ranked DEGs. (C–E) Gene Ontology (GO) Biological Process, Cellular Component, and Molecular Function terms enriched among brown-module genes (top terms; FDR <0.05). (F) KEGG pathways enriched (FDR <0.05); bubble size indicates gene count and color denotes −log10(FDR). Abbreviations: DEG, differentially expressed gene; GO, Gene Ontology; KEGG, Kyoto Encyclopedia of Genes and Genomes; FDR, false discovery rate.

Differential expression analysis of The GEO dataset (GEO: GSE51575) identified 1,178 genes between EBVaGC and EBV-negative GC (EBVnGC), including 658 upregulated and 520 downregulated genes (Figures 1A and 1B). The upregulated genes were primarily enriched in immune response pathways, while the downregulated genes were associated with cell adhesion. Figure 1 provides an overview of discovery (A–B) and enrichment results (C–F).

### Weighted gene co-expression network analysis

**Figure 2:**
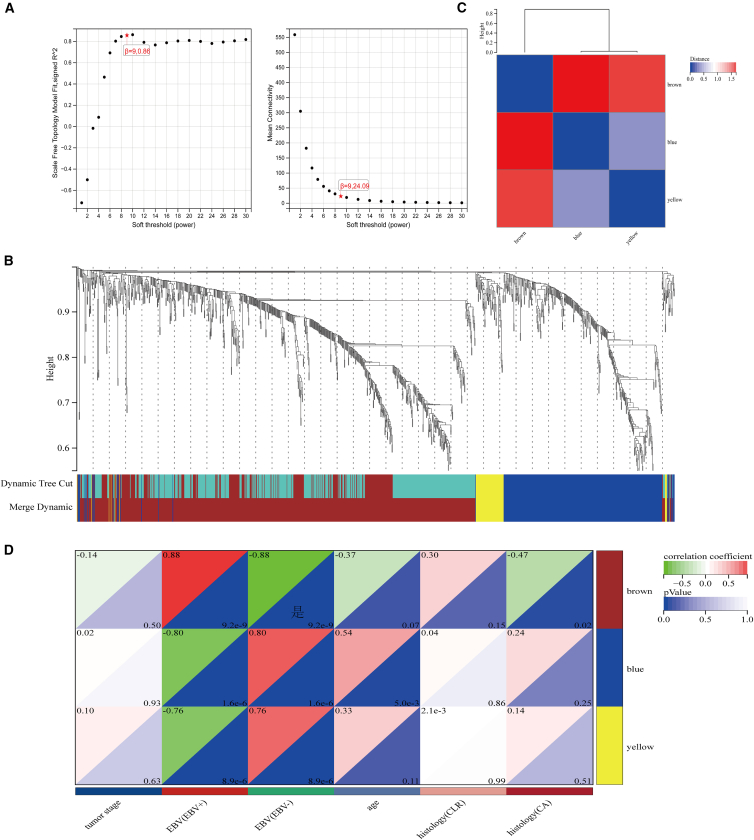
WGCNA of DEGs (A) Selection of soft-thresholding power for scale-free network construction. (B) Hierarchical clustering dendrogram of DEGs with module assignment. Different colors represent distinct co-expression modules identified by WGCNA. (C) Module-trait correlation heatmap shows the association between gene modules and EBVaGC status. Colors represent correlation coefficients from blue (negative) to red (positive). (D) Module-trait relationship matrix displays correlation strength and significance between co-expression modules and EBVaGC.

A soft-thresholding power of 9 was selected to achieve scale-free topology (R^2^ = 0.86, Figure 2A). Dynamic tree cutting identified six distinct gene modules: brown, darkred, greenyellow, lightyellow, black, and lightcyan (Figure 2B). Module-trait correlation analysis revealed varying associations with EBVaGC status (Figure 2C). The brown module demonstrated the strongest positive correlation with EBVaGC and was selected for further analysis (Figure 2D).

### Functional enrichment analysis of the Brown module

To elucidate the biological significance of the brown module, GO and KEGG pathway enrichment analyses were performed using the clusterProfiler package. GO analysis revealed significant involvement in immune-related biological processes, including leukocyte adhesion, immune response activation, T cell activation, and inflammatory response regulation (*p* < 0.05, Figure 1C). Cellular component analysis showed enrichment in plasma membrane structures, endocytic vesicles, and lysosomal membranes (Figure 1D), while molecular function analysis indicated significant enrichment in immune receptor activity, cytokine binding, and chemokine activity (Figure 1E). KEGG analysis demonstrated predominant enrichment in cell adhesion molecules and cytokine-cytokine receptor interaction pathways (*p* < 0.05, Figure 1F), confirming the immunological relevance of the brown module.

### Protein-protein interaction network analysis

A protein-protein interaction (PPI) network was constructed for genes in the brown module via the STRING database and visualized using Cytoscape (Figure S1A). Hub gene analysis based on the degree algorithm (cytoHubba plugin) identified 30 highly connected genes forming 435 interaction edges, with an average clustering coefficient of 0.847. PTPRC (degree = 59) and ITGB2 (degree = 51) ranked as the top two hub genes by degree centrality, underscoring their central regulatory positions within the immune-associated network (Figure S1B). Other top-ranking hub genes included IL10RA, SELL, CD8A, and ITGAL (degrees 48–37), most of which encode immune cell surface receptors or signaling molecules. The prominent network centrality of PTPRC and ITGB2 supports their identification as core immune biomarkers and highlights their potential roles as key regulators in the EBVaGC immune microenvironment (Figure S1C).

### Expression and immune correlation of hub genes

**Figure 3:**
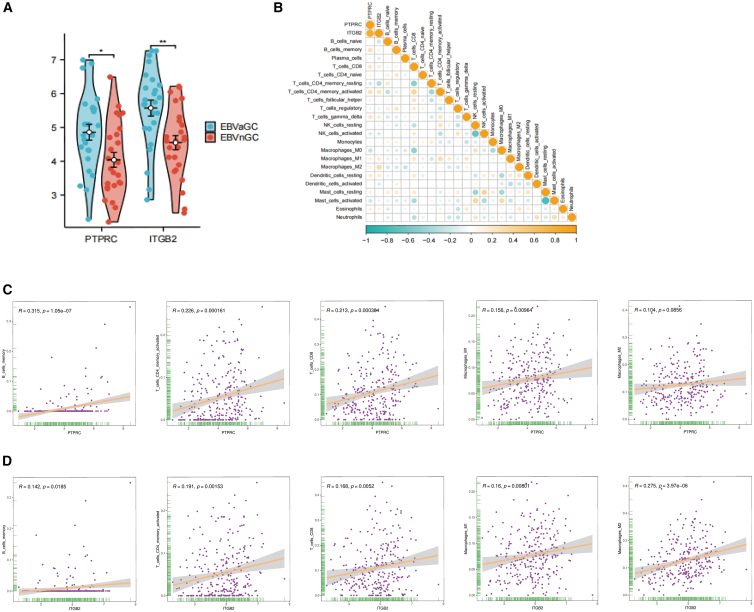
Expression and immune correlation of hub genes (A) Violin plots show the expression levels of PTPRC and ITGB2 in EBVaGC (blue) and EBVnGC (red) from the TCGA-STAD cohort. Statistical significance is indicated (∗*p* < 0.05 and ∗∗*p* < 0.01). (B) Correlation heatmap illustrates the relationships between hub genes (PTPRC and ITGB2) and 22 tumor-infiltrating immune cell subsets. Circle size and color intensity represent the strength of correlation, with orange indicating positive correlations and teal indicating negative correlations. (C) Scatterplots demonstrates correlations between PTPRC expression and infiltration levels of five immune cell types: B cells memory, CD4^+^ memory activated T cells, CD8^+^ T cells, M1 macrophages, and M2 macrophages. Pearson’s correlation coefficients (R) and *p* values are shown for each comparison. (D) Scatterplots show correlations between ITGB2 expression and infiltration levels of the same five immune cell types as in (C). Pearson’s correlation coefficients (R) and *p* values are indicated.

Gene expression analysis based on the TCGA-STAD dataset (25 EBVaGC vs. 249 EBVnGC samples) revealed significantly elevated expression levels of both PTPRC (*p* < 0.05) and ITGB2 (*p* < 0.01) in EBVaGC tissues (Figure 3A). These findings corroborate the network-based identification of PTPRC and ITGB2 as key immune regulatory genes and support their inclusion in subsequent prognostic modeling. To further investigate their potential immunoregulatory roles, we assessed the associations between PTPRC/ITGB2 expression and immune cell infiltration across STAD samples using the CIBERSORT. Both genes exhibited broad positive correlations with various immune subsets, including T cells, B cells, and antigen-presenting cells (Figure 3B), suggesting a coordinated involvement in shaping the tumor immune microenvironment. PTPRC and ITGB2 were significantly correlated with B cells, CD8^+^ T cells, CD4^+^ T cells, and macrophages (Figures 3C and 3D). ITGB2 demonstrated the strongest correlations with M2 macrophage (R = 0.275, *p* = 3.97e−06), and PTPRC showed the strongest association with B cells (R = 0.315, *p* = 1.05e−07), suggesting their involvement in modulating the tumor immune microenvironment in EBVaGC.

### Immunohistochemical validation

To validate the transcriptomic findings at the protein level, immunohistochemical staining was performed to assess the expression and spatial distribution of PTPRC (CD45) and ITGB2 (CD18) in tissue specimens from EBVaGC and EBVnGC (*n* = 20 per group). The analysis focused on quantifying CD45^+^, CD18^+^, and CD68^+^ immune cell densities and their spatial organization within the tumor microenvironment.

**Figure 4:**
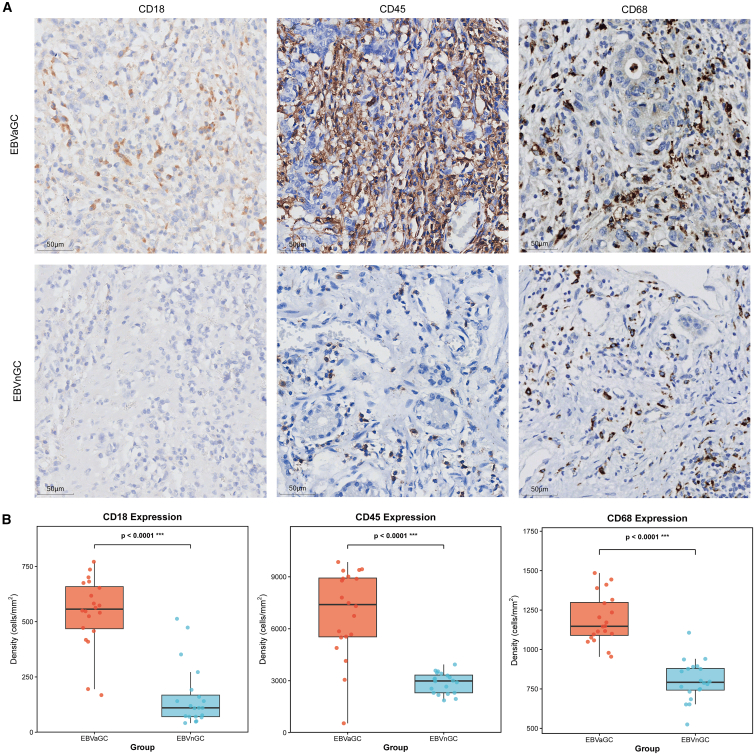
Increased CD18+, CD45+, and CD68^+^ immune-cell infiltration in EBVaGC (A) Representative immunohistochemistry (IHC) micrographs for CD18, CD45, and CD68 in EBVaGC (top) and EBVnGC (bottom). Brown DAB indicates positive staining; hematoxylin counterstain. Images were acquired at 40× objective; scale bars, 50 μm. (B) Dot-and-box plots showing quantitative cell densities (cells/mm^2^) of CD18^+^, CD45^+^, and CD68^+^ cells in EBVaGC versus EBVnGC. Each dot represents one case (EBVaGC *n* = 20; EBVnGC *n* = 20; per case, the mean of ≥3 non-necrotic high-power fields was used). Boxes show IQR, center line the median, whiskers the range. *p* values are from two-sided exact Wilcoxon rank-sum tests (CD18: ∗∗∗*p* < 0.0001; CD45: ∗∗∗*p* < 0.0001; and CD68: ∗∗∗*p* < 0.0001). Units and statistics are indicated on the plots.

Immunohistochemical analysis revealed significantly higher densities of CD45^+^ and CD18^+^ cells in EBVaGC relative to EBVnGC (Figure 4A). CD45^+^ immune cell density was significantly higher in EBVaGC (6907.20 ± 2492.09 vs. 2876.22 ± 594.56 cells/mm^2^, Mann-Whitney U test *p* ≤ 0.0001; Figure 4B; Table S1), with focal clustering in both tumor parenchyma and peritumoral stroma. CD18^+^ cells were also significantly enriched in EBVaGC (541.91 ± 158.83 vs. 159.53 ± 137.90 cells/mm^2^, Mann-Whitney U test *p* ≤ 0.0001; Figure 4B; Table S1), with a predominant localization along tumor-stroma interfaces.

CD68^+^ macrophages were also significantly upregulated in EBVaGC (1189.12 ± 153.99 vs. 802.88 ± 127.59 cells/mm^2^, *p* ≤ 0.0001; Figures 4A and 4B; Table S1). These cells exhibited prominent clustering both in the tumor center and at the tumor-stroma interfaces, aligning with the spatial distribution patterns of CD45^+^ and CD18^+^ cells.

To further characterize the spatial relationships among these immune cell populations, Spearman correlation analysis was performed to assess the strength of association between CD18^+^, CD45^+^, and CD68^+^ cell densities across all samples, providing insight into their potential coordinated distribution within the tumor microenvironment. A strong positive correlation was observed between CD68^+^ macrophages and CD45^+^ lymphocytes (r = 0.744, *p* < 0.001), indicating that macrophages preferentially accumulate in lymphocyte-rich regions. Additionally, a moderate positive correlation was detected between CD68^+^ macrophages and CD18^+^ cells (r = 0.691, *p* < 0.001), suggesting coordinated distribution of these immune cell populations.

Collectively, these immunohistochemical findings demonstrate that the transcriptional upregulation of PTPRC and ITGB2 in EBVaGC is reflected at the protein level and is associated with enhanced immune cell infiltration and spatial reorganization. The correlated distribution patterns of CD68^+^ macrophages, CD45^+^ lymphocytes, and CD18^+^ cells suggest coordinated immune cell recruitment and potential cross-talk that may contribute to the distinct immune landscape of EBVaGC.

### Construction of the immune scoring model

**Figure 5:**
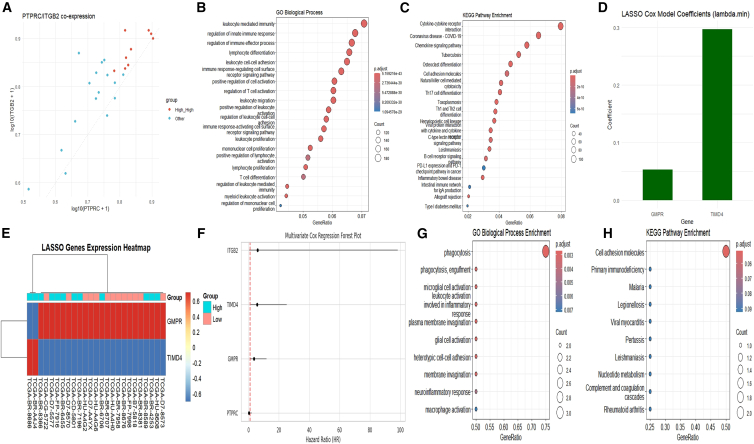
Construction and characterization of the four-gene immune score model in EBVaGC (A) Co-expression analysis of PTPRC and ITGB2 in EBVaGC samples. High-high expression pairs are highlighted in red. (B) GO biological process enrichment of immune genes co-expressed with PTPRC and ITGB2, highlighting terms such as leukocyte-mediated immunity and T cell activation. (C) KEGG pathway enrichment showing cytokine-cytokine receptor interaction, chemokine signaling, and other immune-related pathways. (D) LASSO Cox regression coefficients (lambda.min) for the selected prognostic genes GMPR and TIMD4. (E) Heatmap of GMPR and TIMD4 expression across risk groups. (F) Forest plot of multivariate Cox regression showing hazard ratios (HRs) and 95% confidence intervals for the four model genes. (G) GO enrichment of the final model genes, revealing involvement in phagocytosis, glial cell activation, and inflammatory response. (H) KEGG enrichment of model genes, with pathways such as cell adhesion molecules and primary immunodeficiency.

Building upon these findings, we examined the transcriptional interrelationship between PTPRC and ITGB2 in EBVaGC and observed a strong positive correlation (r = 0.78, *p* < 0.001; Figure 5A). Both PTPRC and ITGB2 are markers of leukocyte abundance, with PTPRC representing a pan-leukocyte marker (CD45) and ITGB2 primarily associated with myeloid-lineage cells, particularly M2 macrophages. As both markers reflect the overall presence of immune cells in the tumor microenvironment, their high correlation is expected, suggesting a parallel increase in leukocyte infiltration and myeloid cell abundance in EBVaGC. Genes co-expressed with either anchor gene (|r| > 0.30) were significantly enriched in leukocyte activation, lymphocyte differentiation, and immune effector functions (GO; Figure 5B), as well as cytokine-cytokine receptor interactions (KEGG; Figure 5C).

Within this immune-related co-expression module, LASSO-penalized Cox regression identified two additional prognostic genes, GMPR (guanosine monophosphate reductase) and TIMD4 (T cell immunoglobulin and mucin-domain containing 4) (Figures 5D and 5E). Together with the two biologically anchored genes PTPRC and ITGB2, these four genes were then used to construct an immune scoring model via multivariable Cox regression in the TCGA EBVaGC cohort. The resulting four-gene Immunoscore was defined as:

Immunoscore=1.2809×GMPR+1.7341×TIMD4−1.1451×PTPRC+1.7869×ITGB2,

where each gene denotes its z-score-standardized expression level (within the EBV-positive TCGA cohort). In this model, GMPR, TIMD4, and ITGB2 act as risk factors (positive coefficients), whereas PTPRC acts as a protective factor (negative coefficient; Figure 5F).

Functional annotation of the co-expression module and of the four-gene set supported the immunological relevance of the Immunoscore, highlighting roles in phagocytosis and cell adhesion pathways (Figures 5G and 5H). We conducted comprehensive sensitivity analyses comparing the purely data-driven two-gene model (GMPR, TIMD4) with the biologically informed four-gene model (GMPR, TIMD4, PTPRC, ITGB2). The gene-level coefficient directions and corresponding hazard ratios for both models are provided in Table S2, including their correlation coefficients. Gene-level coefficient directions and hazard ratios are shown in Figures S2A and S2B. Kaplan-Meier survival analyses demonstrated consistent risk stratification for both models (log rank *p* = 0.0062 for the two-gene model, *p* = 0.0033 for the four-gene model; Figure S2C). Bootstrap-corrected calibration curves at 2.4 years showed comparable predictive accuracy, with slightly improved calibration in the four-gene model (calibration slope: 1.22 vs. 1.38; Figure S2D), confirming that PTPRC and ITGB2 add independent prognostic value beyond GMPR and TIMD4.

### Internal validation and model comparison

**Figure 6:**
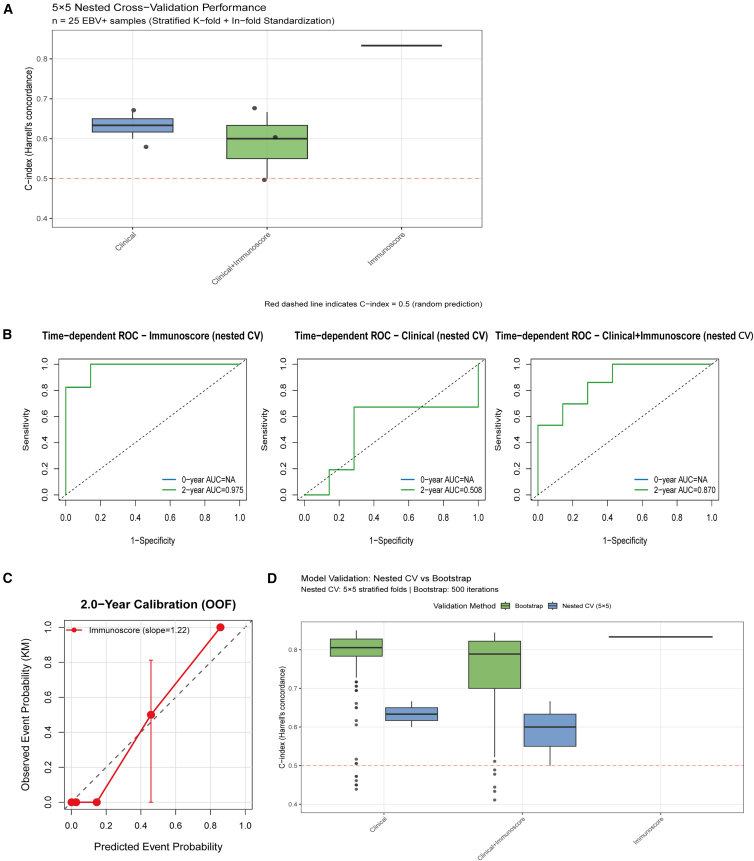
Model validation and predictive performance (A) Nested cross-validation performance. (B) Time-dependent ROC analysis for immunoscore, clinical, and combined models. (C) 2.0-year calibration curve. (D) Comparison of validation methods (nested CV vs. bootstrap).

Following the prespecified 5 × 5 nested cross-validation protocol (event-stratified folds, within-fold standardization, and evaluation restricted to the outer test folds; see method details), we compared three candidate models (Immunoscore, Clinical, and Combined) in the EBV-positive GC cohort (*n* = 25). On the outer test folds, Immunoscore achieved the highest C-index (0.922; 95% CI, 0.755–1.000), whereas Clinical performed worst (0.489; 95% CI, 0.025–0.953); notably, the Combined model did not improve upon Immunoscore (0.622; 95% CI, 0.199–1.000) (Figure 6A; Table S3). The Clinical model incorporated age (continuous) and tumor stage (I/II vs. III/IV), while the Combined model integrated these covariates with the four-gene Immunoscore. No missing data were observed for any variable, as a complete-case analysis was performed (*n* = 25). The inferior performance of the Combined model relative to the Immunoscore alone (C-index: 0.622 vs. 0.922) can be attributed to the statistical penalty incurred from adding non-informative parameters in a small-sample, sparse-event setting (*n* = 25, with 6 events). The contribution of tumor stage was minimal (HR = 0.124, *p* = 0.144), and the incorporation of additional covariates increased model variance without offering a compensatory improvement in model discrimination. This phenomenon is well-documented in statistical literature, particularly when the degrees of freedom approach the number of events. Pairwise ΔC-index values (nested CV) were: Immunoscore − Clinical = +0.433; Combined − Clinical = +0.133; Combined − Immunoscore = −0.300 (Table S4). Time-dependent ROC curves derived from out-of-fold predictions showed clear separation at the adaptively selected 24-month horizon: Immunoscore AUC = 0.975; Combined AUC = 0.870; Clinical AUC = 0.508 (Figure 6B; Table S5). At 24 months, OOF calibration was broadly acceptable (slope = 1.222; intercept = −0.064; 5 risk groups); the slope slightly >1 indicates conservative predictions with mild risk underestimation (Figure 6C; Table S6). Bootstrap optimism-corrected C-indices mirrored the nested-CV ranking (Immunoscore 0.933; Combined 0.869; Clinical 0.814), supporting robustness of the internal validation (Figure 6D). The proportional-hazards assumption was not violated (e.g., immunoscore p = [0.62], stage group p = [0.71], global p = [0.88]); see Table S7 and Figure S3A.

**Figure 7:**
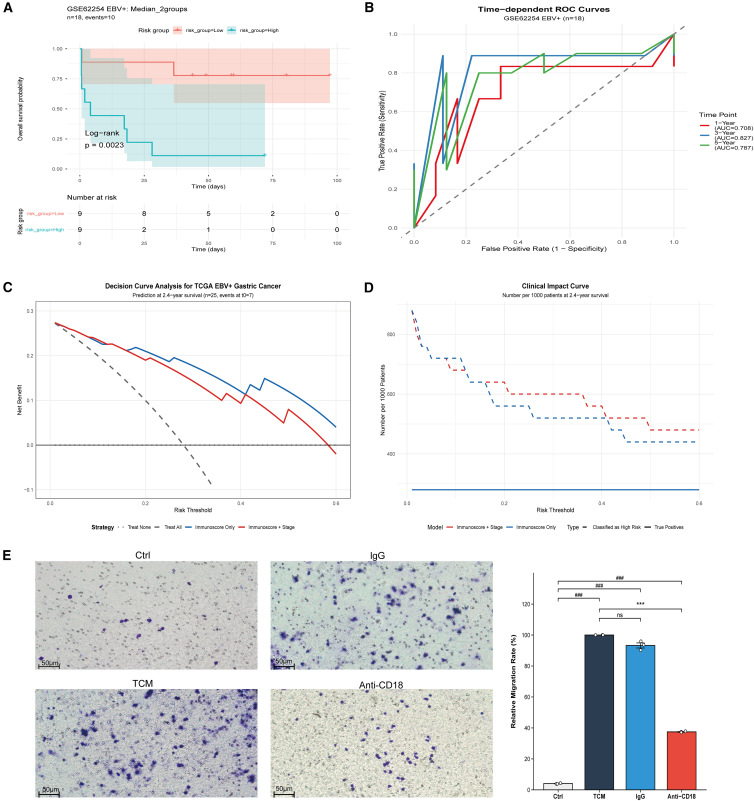
Model validation, clinical utility assessment, and functional validation (A and B) External validation in GSE62254 EBV + GC cohort showing survival stratification and time-dependent ROC curves. (C and D) Decision curve and clinical impact analysis evaluating the added value of immunoscore in TCGA EBV+ GC. (E) Transwell migration assay demonstrates the anti-CD18 inhibitory effect on GC cell migration. Images were acquired at 20× objective; scale bars, 50 μm. (^###^*p* < 0.001 and ∗∗∗*p* < 0.001, ns = not significant).

Across outer folds, the data-driven and biologically informed models showed strong but converging discrimination—1/2/3-year AUCs of 0.907/0.959/0.930 vs. 0.828/0.908/0.919 (Δ: −0.079 to −0.011). (Figures S3B and S3C; Table S8). Direct comparison using median-split stratification confirmed consistent survival separation for both strategies (Figure S2C; two-gene: log rank *p* = 0.0062, HR not estimable due to event sparsity; four-gene: log rank *p* = 0.0033). Calibration performance was similarly robust, with the four-gene model showing marginally better alignment between predicted and observed survival probabilities (Figure S2D). Clinical utility was evaluated separately via decision curve analysis; see section clinical integration and decision analysis (Figures 7C and 7D).

Kaplan-Meier analyses using four prespecified stratification strategies demonstrated consistent risk separation across all approaches. Group sizes were as follows: median split (*n* = 13 vs. 12), tertiles (*n* = 9, 8, 8), quartiles (*n* = 7, 6, 6, 6), and optimal cut-point (*n* = 18 vs. 7). Log rank tests showed significant differences in survival across all stratification schemes: median split (*p* = 0.0033), tertiles (*p* = 1.48 × 10^−5^), quartiles (*p* = 6.14 × 10^−5^), and optimal cut-point (*p* = 1.64 × 10^−6^). Under quartile stratification, the hazard ratio contrasting extreme groups (Q4 vs. Q1) was not reliably estimable due to quasi-complete separation, with very few events observed in the lowest-risk quartile. We therefore rely on Kaplan-Meier curves and log rank tests rather than hazard ratio estimates for this comparison. These sensitivity analyses confirm that our primary conclusions—derived from the continuous immunoscore—remain robust regardless of the chosen categorization approach (Figure S3D; Table S9).

### External validation

External validation in the GSE62254 EBV-positive GC cohort (*n* = 18) demonstrated that the four-gene Immunoscore, applied with fixed TCGA-derived coefficients, retained prognostic discrimination across multiple pre-specified grouping schemes despite the small sample size and limited event count. While these results are promising, it is important to note that the external validation is preliminary and exploratory, and further validation in larger cohorts is needed.

Using a median split (high vs. low), we observed significant prognostic separation (log rank *p* = 0.0023), with a hazard ratio of 8.68 (95% CI: 1.73–43.58) (Figure 7A). Tertile and quartile-based stratification similarly demonstrated significant survival differences (log rank *p* = 0.12,log rank *p* = 0.0226, respectively) (Figures S4A and S4B). The surv_cutpoint-derived two-group solution yielded a hazard ratio of 5.69 (95% CI: 1.17–27.58; *p* = 0.031) (Figure S4D). Time-dependent ROC analysis using the continuous Immunoscore yielded moderate AUC values at clinically relevant horizons, but these estimates are inherently imprecise, given the small number of patients and events (Figure 7B).

At the pre-specified 24-month decision horizon, decision curve analysis based on the continuous score (without refitting) indicated a small positive net benefit over treat-all and treat-none strategies at very low threshold probabilities, with a maximum net benefit of 0.055 at a threshold of approximately 0.01 (C-index = 0.765) (Figures S4E and S4F). However, with only 4 events observed by 24 months (event rate: 22.2%), external DCA and calibration results are presented descriptively without formal hypothesis testing (Figure S4G).

Collectively, these findings support the external transportability of the Immunoscore’s prognostic direction and risk ranking capacity, while explicitly acknowledging the statistical imprecision inherent to the limited external cohort size. The consistency of prognostic signals across multiple grouping strategies and the preservation of discriminative ability (C-index = 0.765) provide preliminary evidence of model generalizability, but further validation in larger, independent EBVaGC cohorts is essential to confirm these findings.

### Clinical integration and decision analysis

To evaluate the incremental clinical utility of integrating conventional staging with molecular stratification, we constructed a parsimonious multivariable Cox regression model including tumor stage (I/II vs. III/IV) and the four-gene Immunoscore. The Immunoscore remained an independent prognostic factor with a substantial hazard ratio (HR = 24.6, 95% CI: 3.12–194.07, *p* = 0.002), whereas stage showed a directionally protective but statistically imprecise and non-significant effect (HR = 0.124, 95% CI: 0.007–2.094, *p* = 0.144), consistent with the limited number of events in the EBV-positive GC subset (Table S10). Decision curve analysis (DCA) at the 24-month (730-day) prediction horizon demonstrated that both the Immunoscore-only and Immunoscore+stage models consistently outperformed treat-all and treat-none strategies across clinically relevant risk thresholds (0.10–0.50). The net-benefit curves were nearly superimposable, with virtually identical peak values (Immunoscore: 0.2347; Immunoscore+stage: 0.2352). Concordantly, Harrell’s C-index was identical for both models (C = 0.933), indicating comparable discriminative performance. Collectively, these results indicate that, in this small EBVaGC cohort, the four-gene Immunoscore captures most of the available prognostic signal, with stage contributing only marginal incremental predictive value (Figures 7C and 7D). Larger, independent cohorts will be required to confirm whether this molecular signature can complement, refine, or potentially subsume conventional anatomic staging in EBVaGC.

### Exploratory functional assay

To functionally interrogate the ITGB2/CD18 axis, we performed THP-1 Transwell chemotaxis assays using EBVaGC-derived tumor-conditioned medium (TCM) as the chemoattractant. Each experimental condition was tested in triplicate wells per experiment, with three independent biological replicates (*n* = 3). Relative migration rates (% of TCM; mean ± SEM) were: Ctrl, 4.03 ± 0.15; TCM, 100.0 ± 0.0; IgG isotype, 93.3 ± 1.64; and anti-CD18, 37.4 ± 0.23. Corresponding migrated-cell counts per field (mean ± SEM) were: Ctrl, 1.53 ± 0.04; TCM, 38.1 ± 0.53; IgG isotype, 35.5 ± 0.88; and anti-CD18, 13.3 ± 0.26 (Figure 7E). One-way ANOVA revealed highly significant differences among groups (F(3,8) = 3054.65, *p* < 0.001). Post-hoc Welch’s t-tests showed that TCM markedly increased THP-1 migration compared to unstimulated control (*p* < 0.001 vs. Ctrl), whereas the IgG isotype control did not significantly differ from TCM (*p* = 0.055), confirming antibody specificity. CD18 blockade significantly reduced migration versus TCM (*p* < 0.001), corresponding to a 62.6% reduction in relative migration rate (100% → 37.4%) and approximately 65% fewer migrated cells per field (38.1 → 13.3) (Figure 7E; Table S11). These findings indicate that the EBVaGC-derived chemotactic recruitment of monocytes is largely dependent on a β_2_-integrin (ITGB2/CD18)-mediated adhesion/migration pathway and provide mechanistic support for the four-gene Immunoscore, in which ITGB2 acts as a risk component. This functional assay is intended as hypothesis-generating for pathway biology rather than a test of clinical predictive performance, which is evaluated in sections internal validation and model comparison and clinical integration and decision analysis.

## Discussion

In the era of precision immuno-oncology, understanding the immune architecture of molecularly distinct tumor subtypes has become paramount for therapeutic stratification. EBVaGC represents a paradigmatic example of virus-driven carcinogenesis with unique immunological features, yet paradoxically remains one of the least characterized subtypes in terms of prognostic biomarkers. This proof-of-concept study addresses this critical knowledge gap by providing the first systematic analysis of immune regulatory networks in EBVaGC, with implications for both biological understanding and clinical translation. Our key contributions include: (1) identification of PTPRC and ITGB2 as central immune hubs orchestrating the distinctive immune landscape of EBVaGC; (2) multi-platform validation of their differential expression across independent transcriptomic datasets; (3) demonstration of their functional associations with macrophage infiltration through bioinformatic inference, immunohistochemical and functional validation; and (4) proof-of-concept that immune-based signatures can achieve meaningful risk stratification in EBVaGC, establishing scientific rationale for larger validation efforts.

Our comprehensive transcriptomic analysis identified 1,178 differentially expressed genes between EBVaGC and EBVnGC, with WGCNA revealing immune-related modules significantly associated with EBVaGC status. Network analysis identified PTPRC and ITGB2 as central hub genes with high connectivity. Transcriptomic validation using TCGA data confirmed significantly elevated expression of both genes in EBVaGC. Immunohistochemical staining further supported these findings, revealing significantly increased infiltration of both CD45^+^(PTPRC) cells and CD18^+^(ITGB2) cells in EBVaGC specimens, with CD45^+^ cell density increasing by 2.4-fold (*p* ≤ 0.0001) and CD18^+^ cells showing a 3.4-fold enrichment (*p* = 0.0355) in EBVaGC relative to EBVnGC. The spatial co-localization of CD45^+^ lymphocytes, CD18^+^ cells, and CD68^+^ macrophages, evidenced by strong correlation coefficients (r = 0.744 and r = 0.691, respectively), suggests coordinated immune cell recruitment and active cross-talk within the tumor microenvironment. Notably, CD68^+^ macrophages were significantly upregulated in EBVaGC (1.5-fold, *p* ≤ 0.0001) and exhibited clustering patterns at tumor-stroma interfaces, aligning with the spatial distribution of CD45^+^ and CD18^+^ cells. This organized immune infiltration pattern may indicate functional specialization, where CD45^+^ lymphocytes engage tumor cells in the parenchyma, while ITGB2-mediated adhesion facilitates immune cell trafficking at the tumor periphery, collectively shaping the distinct immune landscape of EBVaGC.

PTPRC (CD45) is a canonical regulator of T and B cell receptor signaling broadly expressed across leukocytes.^11^ It modulates T cell activation, lymphocyte survival, and cytokine responses through protein tyrosine kinase regulation.^12^^,^^13^^,^^14^ Its immunological role is context-dependent, with dual involvement in immune activation and tolerance.^15^^,^^16^ While prognostic implications vary across cancer types,^17^ PTPRC showed a strong positive correlation with B memory cells infiltration in our study, suggesting its role in promoting immune activation in EBVaGC. However, PTPRC exhibited a negative coefficient in our prognostic model, which might initially seem counterintuitive for a pan-leukocyte marker. This paradox could be explained by the complex, context-dependent role of tumor-infiltrating immune cells: while overall leukocyte abundance (marked by CD45) is associated with immune engagement, the specific immune cell composition and their functional states in the tumor microenvironment play a critical role in clinical outcomes.^18^ In EBVaGC, PTPRC expression may preferentially mark immune cells with anti-tumor activity, such as cytotoxic T lymphocytes and NK cells. However, the higher PTPRC expression could also reflect an immune microenvironment that, despite its apparent immune activation, is ineffective or even detrimental due to the dominance of immunosuppressive mechanisms. Specifically, ITGB2, with a positive coefficient, may predominantly reflect myeloid-lineage cells, including M2 macrophages, which are associated with poor prognosis and immune suppression in EBVaGC.^19^^,^^20^ ITGB2 (CD18) encodes the β2 integrin chain essential for leukocyte adhesion, migration, and signal transduction.^21^ In GC, ITGB2-containing complexes activate TGF-β signaling and extracellular matrix remodeling.^22^ High ITGB2 expression associates with increased tumor aggressiveness across various malignancies.^23^^,^^24^^,^^25^^,^^26^^,^^27^^,^^28^ In our analysis, ITGB2 was markedly upregulated in EBVaGC and correlated with M2 macrophage infiltration, suggesting involvement in immune evasion.^18^

Under a unified 24-month decision horizon, we integrated the assessment of discrimination, calibration, and clinical net benefit. Internally, the four-gene Immunoscore achieved strong discrimination (optimism-corrected C-index = 0.933) and showed nearly identical DCA performance to the “Immunoscore+stage” model (peak net benefit 0.2352 vs. 0.2347, respectively; Figure S3), indicating that the molecular signature captures most staging information in this cohort (Table S2). Although nested cross-validation yielded a C-index of 0.922 (95% CI: 0.755–1.000), this estimate remains imprecise due to the limited number of events, and bootstrap optimism-correction confirmed potential overfitting.

In external validation (GEO: GSE62254 EBV-positive, *n* = 18), the continuous Immunoscore—applied without refitting—retained moderate discrimination (C-index = 0.765) and yielded a small positive net benefit at very low threshold probabilities (maximum net benefit 0.055 at a threshold ≈0.01; Figure S4). No additional tuning or re-weighting was performed; only within-cohort z-standardization was applied to mitigate scale differences, with TCGA-derived coefficients used directly. Because of distributional shifts, the TCGA-derived optimal cut-point failed externally (classifying nearly all samples as high-risk). Consequently, categorical groupings (median, quartiles, and ‘surv_cutpoint’ with ‘minprop = 0.20’) were used solely for visualization and sensitivity analyses, with all primary inferences based on the continuous score. Given that only 4 events were observed by 24 months (22.2%), external DCA and calibration are reported descriptively without formal hypothesis testing.

Overall, concordant directional effects and risk rankings between internal and external analyses support the transportability of the Immunoscore, although the stability of net-benefit estimates will require confirmation in larger independent cohorts. The preservation of discriminative ability in GSE62254 (C-index = 0.765) and similar event rates between cohorts (22.2% vs. 24.0% in TCGA) provide preliminary evidence of generalizability, while underscoring the statistical imprecision inherent to small EBVaGC samples.

*In vitro* validation provides preliminary mechanistic evidence for ITGB2’s role in immune cell recruitment. CD18 blockade reduced THP-1 migration by 62.6% (*p* < 0.001) while IgG control showed no effect, confirming ITGB2-mediated adhesion as a key mechanism. This aligns with ITGB2’s positive prognostic coefficient, suggesting enhanced ITGB2-dependent infiltration may associate with adverse outcomes, potentially through the recruitment of immunosuppressive populations such as M2 macrophages. The spatial co-localization of CD18^+^ and CD68^+^ cells supports this interpretation. However, these findings are hypothesis-generating rather than definitive, as THP-1 cells may differ from primary immune cells in function and migration patterns, and the short-term assay (6 h) cannot recapitulate *in vivo* complexity. Further validation using primary patient-derived immune cells, spatial transcriptomics, and *in vivo* models is essential to establish causality and assess ITGB2 as a therapeutic target for improving outcomes in EBVaGC.

Positioning within the landscape of GC immune signatures. Our four-gene Immunoscore addresses a distinct niche compared with established immune-based prognostic tools in gastric cancer. The Galon Immunoscore, which quantifies CD3^+^ and CD8^+^ T cell densities at the tumor center and invasive margin, has been validated primarily in colorectal cancer and requires standardized immunohistochemistry with spatial assessment protocols that may limit scalability.^29^ Tumor-infiltrating lymphocyte (TIL) scoring, while prognostically relevant in GC broadly, relies on subjective morphological assessment and does not capture the myeloid-macrophage axis central to EBVaGC biology. Among transcriptome-based classifiers, the Tumor Immune Dysfunction and Exclusion (TIDE) score predicts immunotherapy response across tumor types but was not developed for EBVaGC-specific risk stratification.^30^ The Asian Cancer Research Group (ACRG) molecular classification identifies immune-enriched subtypes with a favorable prognosis, yet this framework encompasses all GC rather than addressing EBV-specific heterogeneity.^10^^,^^31^ Other published signatures—including the immunotype-based consensus molecular subtypes—provide valuable biological stratification but lack the specificity required for clinical decision-making within the relatively rare EBVaGC population. Our signature represents a conceptually distinct approach: rather than applying pan-GC immune metrics to an EBV-positive subset, we derived features directly from EBVaGC-enriched transcriptomic modules, capturing not only lymphocyte abundance (PTPRC) but also myeloid adhesion biology (ITGB2), purine metabolism (GMPR), and TIM-4-mediated phagocytic clearance (TIMD4). This EBVaGC-specific derivation may explain the strong internal discrimination (C-index = 0.922), though direct head-to-head performance comparisons against alternative signatures await larger validation cohorts. Importantly, our findings are hypothesis-generating rather than practice-changing: they establish biological plausibility for subtype-specific prognostication in EBVaGC and provide a rationale for international collaborative validation efforts. The selective elevation of PTPRC and ITGB2 in EBV-associated tumors suggests that viral infection modulates this immunoregulatory axis, though direct mechanistic relationships warrant further investigation.^8^^,^^32^^,^^33^^,^^34^

From a translational medicine perspective, this proof-of-concept investigation addresses a critical gap in rare cancer precision medicine. EBVaGC represents approximately 10% of patients with GC yet lacks subtype-specific prognostic tools, with current practice applying generic protocols. Our findings provide the first systematic evidence that EBVaGC harbors sufficient immune heterogeneity to support dedicated biomarker development, transforming this from an untested hypothesis to a biologically supported research priority. The identification of PTPRC and ITGB2 as central immune regulators offers both prognostic and potential therapeutic implications. Notably, EBVaGC is characterized by high PD-L1 expression and extensive T cell infiltration, making it a promising candidate for immune checkpoint inhibitor (ICI) therapy. Recent clinical trials have demonstrated that PD-1/PD-L1 inhibitors, such as pembrolizumab and nivolumab, achieve durable responses in a subset of patients with EBVaGC, with objective response rates reaching 100% in some small cohorts. Our four-gene Immunoscore, by capturing the balance between immune activation (PTPRC-marked lymphocytes) and immunosuppression (ITGB2-associated myeloid infiltration), may help identify patients most likely to benefit from ICI therapy. Furthermore, the functional role of ITGB2 in macrophage recruitment suggests that combination strategies targeting both checkpoint molecules and myeloid-mediated immunosuppression could enhance therapeutic efficacy in patients with high-risk EBVaGC. While clinical implementation requires substantial validation, this study establishes a scientific rationale for international collaborative validation efforts in this underserved patient population.

This proof-of-concept study lays the foundation for precision biomarker development in EBVaGC, a distinct GC subtype that remains clinically underserved. By identifying PTPRC and ITGB2 as central immune regulators and demonstrating the feasibility of subtype-specific prognostic modeling, we provide valuable insights into the future of EBVaGC biomarker research. While further validation is necessary, including prospective studies with larger multi-center cohorts (sample size ≥100), these findings contribute to the establishment of a biologically supported research framework. For patients with EBVaGC currently managed under generic protocols, this study provides a scientific foundation for future precision medicine approaches in this important, rare cancer population.

### Limitations of the study

This study has several limitations. First, the sample size is small, with only 25 cases of EBVaGC, and the external validation sample is also limited (*n* = 18). This may affect the statistical precision of the model and the stability of external validation. Although the four-gene Immunoscore showed strong prognostic discrimination in internal validation (C-index = 0.922), the C-index in external validation (GEO: GSE62254) was 0.765, indicating that the model’s generalizability requires further validation. Second, due to the scarcity of samples, there may be a risk of data overfitting in the EBVaGC study, despite optimization corrections. Additionally, while the functional validation of immune features was provided, the use of the THP-1 cell line and short-term *in vitro* experiments may not fully reflect the complexity of the *in vivo* immune microenvironment. Therefore, future studies will require more clinical samples, multi-center validation, and the use of more clinically relevant models.

## Resource availability

### Lead contact

Further information and requests for resources and reagents should be directed to and will be fulfilled by the lead contact, Jian-ning Chen (chjning@mail.sysu.edu.cn).

### Materials availability

No new unique materials were generated during this study. All existing materials can be requested from the corresponding author.

### Data and code availability

• Data: The publicly available datasets analyzed in this study are available from TCGA-STAD (https://portal.gdc.cancer.gov) and GEO (accession numbers GSE51575 and GSE62254; see key resources table). De-identified IHC quantification data, per-sample immunoscores, and sample ID lists are deposited in Mendeley Data (https://doi.org/10.17632/tb673mfw47.2).
• Code: Minimal R scripts to reproduce key analyses, including the four-gene Immunoscore formula (Immunoscore = 1.2809 × GMPR + 1.7341 × TIMD4 − 1.1451 × PTPRC + 1.7869 × ITGB2) and exact model coefficients, are deposited in Mendeley Data (https://doi.org/10.17632/tb673mfw47.2).
• Any additional information required to reanalyze the data reported in this article is available from the lead contact upon request.

## Acknowledgments

This work was supported by the 10.13039/501100001809National Natural Science Foundation of China (82403764), 10.13039/501100002858China Postdoctoral Science Foundation (2022M713568 and 2023M744005), the 10.13039/501100021171Guangdong Basic and Applied Basic Research Foundation (2024A1515012426 and 2022A1515012555), and the 10.13039/501100010256Guangzhou Municipal Science and Technology Project (202102010156).

## Author contributions

Conceptualization, R.H., R.Y., and J.C.: data analysis, R.H., R.Y., and Z.F.; interpretation of data, R.H., R.Z., Z.F., and B.L.; collection of clinical specimens, Y.G., S.L., R.Z., D.Y., and C.S.; investigation, Y.G., S.L., Y.P., J.G., and R.Z.; immunochemical staining, R.H., Y.P., J.G., D.Y., and C.S.; writing – original draft, R.H.; writing – review and editing, J.C.; supervision: Y.D., Y.S., and J.C.; funding acquisition: Y.D., J.C., Y.S., and C.S. All authors reviewed and approved the final article.

## Declaration of interests

The authors declare no competing interests.

## STAR★Methods

### Key resources table

**Table:** 

REAGENT or RESOURCE	SOURCE	IDENTIFIER
**Antibodies**		

Anti-CD45 (PTPRC), mouse monoclonal, clone PD7/26+2B11	Zhongshan Golden Bridge Biotechnology (ZSGB-BIO)	Cat# Kit-0024
Anti-CD18 (ITGB2), rabbit monoclonal	Abcam	Cat# ab307406
Anti-CD68, mouse monoclonal, clone KP1	ZSGB-BIO	Cat# ZM-0060
Anti-CD18 (ITGB2) blocking antibody, mouse	BioLegend	Cat# 302102; RRID: AB_314218
Mouse IgG1 κ isotype control	BioLegend	Cat# 400102; RRID: AB_326462

**Biological samples**		

Human GC FFPE tissues (EBVaGC, n=20)	Third Affiliated Hospital, Sun Yat-sen University	Ethics approval: RG2023-144-02
Human GC FFPE tissues (EBVnGC, n=20)	Third Affiliated Hospital, Sun Yat-sen University	Ethics approval: RG2023-144-02

**Chemicals, peptides, and recombinant proteins**		

Novolink Polymer Detection System	Leica Biosystems	Cat# RE7280-K
DAB (3,3’-Diaminobenzidine) Substrate Kit	ZSGB-BIO	Cat# ZLI-9018
Hematoxylin	ZSGB-BIO	Cat# ZLI-9610
Crystal Violet Solution	Sigma-Aldrich	Cat# V5265
EDTA Antigen Retrieval Solution (pH 8.0)	ZSGB-BIO	Cat# ZLI-9066
EDTA Antigen Retrieval Solution (pH 9.0)	ZSGB-BIO	Cat# ZLI-9068
Citrate Antigen Retrieval Solution (pH 6.0)	ZSGB-BIO	Cat# ZLI-9064
RPMI 1640 Medium	Gibco	Cat# 11875093
Fetal Bovine Serum (FBS)	Gibco	Cat# 10099141C

**Critical commercial assays**		

Transwell Permeable Supports, 8.0 μm pore, 24-well	Corning	Cat# 3422
Millex-GV Syringe Filter, 0.22 μm	Millipore	Cat# SLGV033RS

**Deposited data**		

GSE51575 (Discovery cohort)	GEO	GSE51575
TCGA-STAD RNA-seq (Training cohort)	TCGA	https://portal.gdc.cancer.gov
GSE62254 (External validation cohort)	GEO	GSE62254
Platform annotation	GEO	GPL13607
Four-gene Immunoscore model coefficients and per-sample scores	Mendeley Data	https://doi.org/10.17632/tb673mfw47.2

**Experimental models: Cell lines**		

THP-1 (human monocytic leukemia)	ATCC	Cat# TIB-202; RRID: CVCL_0006
AGS-EBV (EBV-positive GC)	Laboratory stock	N/A

**Software and algorithms**		

R (version ≥4.3)	R Project	https://www.r-project.org; RRID: SCR_001905
limma (v3.40.6)	Bioconductor	RRID: SCR_010943
WGCNA	CRAN	RRID: SCR_003302
clusterProfiler	Bioconductor	RRID: SCR_016884
glmnet	CRAN	RRID: SCR_015505
survival	CRAN	RRID: SCR_021137
survminer	CRAN	N/A
survcomp	Bioconductor	N/A
timeROC	CRAN	N/A
rms	CRAN	RRID: SCR_016352
rmda	CRAN	N/A
ggplot2	CRAN	RRID: SCR_014601
Cytoscape	Cytoscape Consortium	https://cytoscape.org; RRID: SCR_003032
cytoHubba	Cytoscape App Store	N/A
STRING (v11.5)	STRING Consortium	https://string-db.org; RRID: SCR_005223
TIMER 2.0	Cistrome	https://cistrome.shinyapps.io/timer/
CIBERSORT	Stanford University	https://cibersort.stanford.edu; RRID: SCR_016955
ImageJ (v1.53)	NIH	RRID: SCR_003070
Panoramic 250 FLASH Digital Pathology Scanner	3DHISTECH Ltd	N/A

**Other**		

MSigDB C7 immunologic gene sets	Broad Institute	https://www.gsea-msigdb.org

### Experimental model and study participant details

#### Ethics approval

This study was approved by the Ethics Committee of The Third Affiliated Hospital of Sun Yat-sen University under the ethics approval number RG2023-144-02, which specifically covers all aspects of the current research. All human tissue samples were collected with written informed consent from patients, and all procedures involving human participants were conducted in accordance with the principles of the Declaration of Helsinki. The use of publicly available, de-identified data from The Cancer Genome Atlas (TCGA) and the Gene Expression Omnibus (GEO) was reviewed by the same ethics committee, which confirmed that such analyses are exempt from additional ethical approval.

#### Human subjects

Formalin-fixed, paraffin-embedded (FFPE) tissue samples from 20 EBVaGC and 20 EBVnGC patients were obtained from the Department of Pathology, The Third Affiliated Hospital of Sun Yat-sen University (collected between January 2017 and December 2025). Ethical approval was granted by the hospital's Ethics Committee (Approval No. RG2023-144-02). All patients provided written informed consent, and all procedures were conducted in accordance with the Declaration of Helsinki. The cohort comprised 11 males and 9 females, with a median age of 59 years (range: 35–86 years). All patients were of Han Chinese ancestry. Due to the limited sample size, sex-stratified analyses were not performed; the potential influence of sex on the study results cannot be excluded and represents a limitation of this study.Clinical characteristics of the patient cohort are summarized in Table S1.

#### Cell lines

THP-1 cells (*Homo sapiens*; acute monocytic leukemia; male; ATCC Cat# TIB-202) are a human monocytic cell line carrying a translocation t(9;11)(p22;q23) and were maintained in RPMI-1640 supplemented with 10% FBS at 37°C with 5% CO_2_. AGS-EBV cells (*Homo sapiens*; gastric adenocarcinoma; female; latently infected with EBV type 1, harboring episomal EBV genomes with a latency II/III gene expression pattern) were maintained as laboratory stock and cultured under the same conditions. Cell line identity was confirmed by STR profiling, and mycoplasma contamination was routinely tested.

#### Public datasets

The GEO dataset (GEO: GSE51575) was derived from a case-control transcriptomic study by Kim et al.^35^ (Gastroenterology 2015) investigating immune response gene deregulation in Korean EBVaGC patients, comprising matched tumor and adjacent normal tissues profiled on the Affymetrix Human Gene 1.0 ST platform (12 EBVaGC vs. 50 EBVnGC). TCGA: TCGA-STAD RNA-seq data were obtained as TPM-normalized values (25 EBVaGC vs. 249 EBVnGC classified per established criteria). The GEO dataset (GEO: GSE62254) served as the external validation cohort (18 EBV-positive GC cases, Cristescu et al. 2015).

### Method details

#### Data acquisition and processing

Expression data from The GEO dataset (GEO: GSE51575) were retrieved from the Gene Expression Omnibus (GEO) database. Probe identifiers were converted to gene symbols using the GPL13607 platform annotation file. Expression values were log_2_-transformed and quantile-normalized within the dataset using R. TCGA RNA-seq data (TCGA-STAD) were obtained as TPM-normalized gene expression values, with subsequent log_2_(TPM+1) transformation applied. Since each dataset was analyzed independently within its respective cohort, batch effects between datasets were not directly corrected. Within-cohort z-score standardization was applied in the external cohort (GEO: GSE62254) before analysis to minimize platform-specific scale differences while preserving relative expression patterns.

#### Differential expression analysis

Differential gene expression analysis was conducted using the limma R package [version 3.40.6]. Genes with adjusted P < 0.05 and |log_2_FC| > 1 were considered significantly differentially expressed.

#### Weighted gene co-expression network analysis

WGCNA was employed to construct co-expression modules from DEGs.^36^ A scale-free network topology was achieved using Pearson correlation with soft-thresholding power of 9. Modules were defined with minimum size of 30 genes and merged at cut height 0.25, ultimately yielding six distinct modules. This subset-based approach was adopted to focus on genes with demonstrated differential expression between EBVaGC and EBVnGC, thereby reducing computational burden and excluding genes with minimal variance that are unlikely to form meaningful co-expression modules, consistent with established practice in cancer transcriptomic studies.

#### Functional enrichment analysis

Gene Ontology (GO) and Kyoto Encyclopedia of Genes and Genomes (KEGG) pathway enrichment analyses were performed on the EBVaGC-associated module using clusterProfiler.^37^ Statistical significance for enrichment was set at a P-value < 0.05.

#### Hub gene identification

Protein-protein interaction networks were constructed using STRING database (version 11.5) and subsequently visualized with Cytoscape software.^38^ Hub genes were identified using the Degree algorithm in cytoHubba.^39^ The top 30 genes exhibiting the highest connectivity were selected as hub genes.

#### TCGA validation

The expression of identified hub genes was validated using publicly available data from TCGA, analyzing 25 EBVaGC and 249 EBVnGC cases classified based on established criteria.^40^ Expression levels were visualized as violin plots using R software.

#### Tumor immune score analysis

Six distinct tumor-infiltrating immune cell types were quantified using data obtained from the Tumor Immune Estimation Resource (TIMER 2.0) (https://cistrome.shinyapps.io/timer/). Immune cell deconvolution was performed using CIBERSORT-ABS with the LM22 signature matrix (1,000 permutations) for absolute immune cell fraction estimation. Between-group comparisons of immune cell infiltration levels between EBVaGC and EBVnGC were performed using the Wilcoxon rank-sum test. Correlations between continuous hub gene expression levels and estimated immune cell infiltration scores within the EBVaGC cohort were assessed using Spearman's rank correlation coefficient. Multiple testing correction was applied using the Benjamini-Hochberg method, with false discovery rate (FDR) < 0.05 considered statistically significant. FDR-adjusted P values for key correlations are provided in the supplemental information.

#### Immunohistochemistry and quantitative analysis

Formalin-fixed, paraffin-embedded (FFPE) tissue samples from 20 EBVaGC and 20 EBVnGC patients, collected between January 2017 and December 2025, were obtained from the tissue bank of The Third Affiliated Hospital of Sun Yat-sen University. Clinical characteristics of the patient cohort are summarized in Table S1. Ethical approval for this study was granted by the hospital’s Ethics Committee (Approval No. RG2023-144-02). For CD45 (ZSGB-BIO, mouse monoclonal, clone PD7/26+2B11; Kit-0024, dilution 1:300), antigen retrieval was performed in EDTA buffer (pH 8.0) using microwave treatment, followed by primary antibody incubation at 37 °C for 40 minutes. For CD18 (Abcam, ab307406, dilution 1:100), antigen retrieval was conducted in EDTA buffer (pH 9.0) with overnight incubation at 4 °C. For CD68 staining (ZSGB-BIO, mouse monoclonal, clone KP1; ZM-0060, dilution 1:200), antigen retrieval was performed in citrate buffer (pH 6.0) using microwave treatment, followed by primary antibody incubation at 37°C for 40 minutes. Endogenous peroxidase activity and nonspecific binding were blocked using standard reagents. Sections were then incubated with reagents from the Novolink™ Polymer Detection System (Leica Biosystems) and visualized using 3,3′-diaminobenzidine (DAB) chromogen. Slides were counterstained with hematoxylin and mounted. Negative controls were prepared by omitting the primary antibody.

Slides were digitized at 40× magnification using an automatic digital slide scanner (Panoramic 250 FLASH, 3DHISTECH Ltd, Budapest, Hungary). Color deconvolution was performed in ImageJ (v1.53, NIH, bundled with Java 8) to isolate the DAB channel.^41^ Immunopositive cells were identified by the presence of brown cytoplasmic or membranous DAB staining. Regions of interest (ROIs) representing tumor parenchyma and adjacent stroma were manually defined, excluding areas of necrosis, hemorrhage, and large vessels. Five non-overlapping high-power fields (HPFs; 400× magnification, 0.0755 mm^2^ each) were randomly sampled per case. Positive cells were quantified using intensity thresholding, and cell density was expressed as cells per square millimeter (cells/mm^2^).^42^^,^^43^

#### Transwell migration assays

THP-1 chemotaxis toward AGS-EBV–derived tumor-conditioned medium (TCM) was evaluated in 24-well Transwells (8-μm pores) using RPMI-1640. AGS-EBV cells were cultured to 80–90% confluence, switched to serum-free RPMI-1640 for 24 h, and the 0.22-μm–filtered supernatant was used as TCM. THP-1 cells were washed, resuspended in serum-free RPMI-1640 (1×10ˆ6 cells/mL), preincubated for 30 min with anti-CD18 (10 μg/mL) or IgG control, and 2×10ˆ5 cells were added to the upper chamber; lower chambers contained RPMI-1640 with 5% FBS ± 50% TCM. After 6 h, non-migrated cells were removed, migrated cells on the lower membrane surface were fixed, crystal-violet stained, and counted microscopically; migration was normalized to control and compared by one-way ANOVA.

#### Construction of the immune scoring model

Given the rarity of EBVaGC, model development was based on the 25 TCGA EBV-positive GC cases and should be regarded as proof-of-concept rather than a definitive clinical tool. Candidate features were first derived from MSigDB C7 immunologic gene sets. For biological anchoring, PTPRC (CD45; pan-leukocyte identity) and ITGB2 (β2-integrin; leukocyte adhesion) were pre-specified *a priori*. An immune-related module was formed by genes showing Spearman correlation with either anchor (|r| > 0.30), and its functional relevance was confirmed by GO and KEGG enrichment analysis (clusterProfiler).

Two complementary construction strategies were evaluated to control selection bias. (i) Purely data-driven: LASSO-penalized Cox regression with gene selection performed in the inner loop of the nested cross-validation. The regularization parameter λ was selected using 10-fold cross-validation to minimize cross-validation error (lambda.min = 0.294). Given the small sample size (n=25) and high-dimensional feature space, a relatively strong regularization was required to prevent overfitting. This approach consistently identified a sparse two-gene signature (GMPR, TIMD4) with stable coefficients across nested cross-validation folds. (ii) Biologically informed: the two anchors (PTPRC, ITGB2) were forced into the multivariable model by setting `glmnet::penalty.factor = 0` for these genes while penalizing the remaining candidates, producing a four-gene score (GMPR, TIMD4, PTPRC, ITGB2).

For both strategies, predictors were z-standardized within the training folds only; coefficients were then frozen and applied unchanged to the corresponding outer-test folds and to external validation, thereby avoiding information leakage. The final immunoscore was computed as the z-score–standardized linear predictor of the four genes, with coefficients fixed from the training cohort. Performance estimation (5×5 nested cross-validation with event-stratified folds), time-dependent ROC analyses, calibration, decision-curve analysis, and proportional-hazards checks are detailed in the following sections: internal validation and quantification and statistical analysis.

#### Internal validation

Internal validation was performed using 5×5 nested cross-validation with event-stratified splits in both the outer and inner loops. To prevent information leakage, continuous predictors were z-score standardized within each outer training fold, and the same transformation parameters were applied to the corresponding outer test fold. The inner 5-fold loop was used to tune the regularization parameter λ for the LASSO model (Strategy 1); for the fixed four-gene model (Strategy 2), the inner loop was mirrored without hyperparameter tuning to maintain identical data partitioning. Unbiased performance estimates were computed exclusively on outer test folds and aggregated across all folds.

The primary discrimination metric was Harrell’s C-index, summarized as the mean across outer folds with 95% confidence intervals derived from fold-level standard errors. Using out-of-fold (OOF) linear predictors, we computed time-dependent ROC curves and AUC values at nominal horizons of 1, 3, and 5 years. When statistical information was insufficient at a given horizon, a pre-specified fallback time point (e.g., 24 months) was applied based on risk set size and event-count thresholds; these common horizons were determined once on the full dataset and then applied uniformly across all models.

OOF calibration was assessed by grouping patients into predicted-risk quantiles and comparing model-predicted probabilities with Kaplan–Meier–derived observed event probabilities; calibration intercept and slope were reported.

Bootstrap validation used B = 1,000 resamples in the final analyses (developmental piloting used B = 500) and reported optimism-corrected C-indices. In bootstrap analyses—unlike nested cross-validation—predictors were standardized separately within each bootstrap sample and within the corresponding original-sample test set to prevent leakage. The proportional hazards (PH) assumption was tested using cox.zph with the Kaplan–Meier transform; term-wise and global test results, along with Schoenfeld residual plots, are provided in the supplemental information. Source data include per-sample outer-test predictions and a complete validation report.

#### External validation

External validation was performed in the EBV-positive GC subset of GSE62254, with EBV status determined from the original clinical annotations. For each EBV-positive sample, we computed the four-gene Immunoscore as a fixed linear combination of GMPR, TIMD4, PTPRC, and ITGB2 using coefficients derived from the TCGA cohort, without any refitting or re-weighting. To account for cross-study scale differences, raw scores were z-standardized within the external cohort (mean = 0, SD = 1) prior to analysis.

Because the TCGA-derived optimal cut-point did not transfer to GSE62254 (all samples were classified as “high” under this threshold), we employed pre-specified distribution-based grouping schemes for visualization and robustness assessment: (i) median split (High vs. Low), (ii) tertiles, (iii) quartiles, and (iv) an optimal two-group cut-point estimated using surv_cutpoint with minprop = 0.20 to prevent extreme imbalance. Importantly, all primary inferential statements were based on the continuous Immunoscore; these categorical groupings were used for display and sensitivity analyses only.

External validation analyses included Kaplan–Meier survival curves with log-rank tests (for each grouping scheme), and time-dependent ROC analysis with AUC calculations at clinically relevant horizons (nominally 1–3 years) using the continuous score; later time points (e.g., 5 years) were omitted when under-powered. Descriptive calibration and decision-curve analyses were performed when sample size and event counts permitted. Overall survival in GSE62254 was analyzed in months; for decision-curve analysis, we used a pre-specified 24-month prediction horizon to align with the internal DCA performed at 24 months (730 days) in the TCGA cohort. Given the limited number of events in the external cohort, no multivariable models were fitted.

### Quantification and statistical analysis

All statistical analyses were performed in R (version ≥4.3). Survival time was measured in months; events were coded as 1 and censored observations as 0. Categorical variables (e.g., tumor stage) were modeled as nominal factors with a pre-specified reference level (earliest stage) and encoded using indicator variables where appropriate.^44^ Except for within-outer-fold standardization employed in nested cross-validation,^45^ all models were fitted exclusively on training data and subsequently applied unchanged to corresponding test or external validation sets, ensuring no information leakage. Analyses were conducted under a complete-case framework.

Harrell’s C-indices were computed using survcomp::concordance.index, with internal consistency checks performed via Hmisc::rcorr.cens reported; concordance.index used for cross-checks. When prespecified time horizons were underpowered, an *a priori* rule was applied: valid time points required ≥10 subjects at risk and ≥5 events (fallback threshold: ≥5 at risk and ≥3 events). Final time horizons are annotated in the figure captions.

Calibration was assessed using OOF predictions grouped by risk quantiles (default: 5 groups; adaptively reduced for small sample sizes), with Kaplan–Meier–based observed probabilities and calibration slope/intercept reported. Decision curve analysis (DCA) was performed using the rmda package. The proportional hazards assumption was evaluated using cox.zph with the Kaplan–Meier transform; global and term-specific test results are provided in the supplemental information.

All statistical tests were two-sided with a significance level of α = 0.05. Given the limited sample size, we emphasize effect sizes and confidence intervals over multiple significance claims. Sensitivity analyses using LASSO-penalized Cox regression were conducted within the same nested cross-validation framework. Key packages used include: survival, timeROC, rms, rmda, glmnet, ggplot2, Hmisc, and survminer. Random seeds are specified in the analysis scripts (e.g., set.seed(2025)).^46^

We evaluated the PH assumption for all Cox models using scaled Schoenfeld residuals (R cox.zph). The Kaplan–Meier transformation was used as the primary test, with rank and identity transformations as sensitivity checks.^47^ For each covariate and for the global model we reported test p-values (two-sided; p<0.05 indicates violation) and visually inspected residual-vs-time plots. All tests showed no evidence of violation. Full results are provided in Table S1 and residual plots in Figure S1.

Risk stratification of the continuous immunoscore was performed solely for visualization and descriptive summaries; it was not incorporated into model training or internal validation. To assess the robustness of risk group definitions, we evaluated four cut-point strategies: (i) median split (High vs. Low), (ii) tertiles, (iii) quartiles, and (iv) a data-driven optimal cut-point determined using the `surv_cutpoint` function with a minimum group proportion constraint of 0.20 to prevent overly imbalanced groups. For each strategy, we reported group sizes, log-rank p-values, and hazard ratios from Cox proportional hazards models comparing the highest versus lowest risk groups. Because primary inference relied on the continuous immunoscore (C-index and time-dependent AUC), these categorical stratifications served as sensitivity analyses to demonstrate that findings were not dependent on specific cut-point or categorization choices. We examined reasonable alternative tuning settings and observed no material changes in discrimination or time-dependent AUC; therefore the parsimonious solution is reported throughout.^31^^,^^48^

For THP-1 Transwell migration assays, each experimental condition was tested in triplicate wells (technical replicates) per experiment, and the entire experiment was repeated independently three times (n=3 biological replicates). For each biological replicate, the mean of technical replicates was used. Migration rates were background-corrected, normalized to the TCM control (100%), and compared using one-way ANOVA followed by Welch's t-tests for pairwise comparisons (two-sided, α = 0.05). Welch's t-test was employed to account for unequal variances across groups. Results are expressed as mean ± SEM of biological replicates.

IHC quantification was performed across five high-power fields (HPFs, 400×) per case, with the median value reported as cells/mm^2^. Data normality and variance homogeneity were assessed using Shapiro–Wilk and Levene's tests. Between-group comparisons were conducted using Student's t-test (or Welch's t-test for unequal variances) or Mann–Whitney U test as appropriate. Multiple comparisons were corrected using the Benjamini–Hochberg method (FDR reported). Effect sizes (Cohen's d or rank-biserial correlation) were calculated with conventional thresholds. All tests were two-tailed with α = 0.05. Complete statistical outputs (including IHC quantifications), analysis scripts, raw HPF images, anonymized sample identifiers, and fitted model coefficients are provided in the supplemental information.

Table S12 summarizes the key methodological parameters used in this study, including data preprocessing, differential expression analysis, and immune infiltration analysis. Detailed descriptions of each parameter can be found in the table. A completed TRIPOD checklist mapping each reporting item to its location in the manuscript is provided in Table S13.

Statistical details for all experiments, including the exact value of n, the statistical tests used, and measures of central tendency and dispersion, are reported in the corresponding figure legends and results section.

### Additional resources

This study did not involve any clinical trials. No clinical trial registry numbers are applicable.

## Supplemental information

Document S1. Figures S1–S4

Table S1. Immunohistochemical Results and Patient Characteristics for Our Immunohistochemical Validation Cohort, related to Figure 4

Table S2. Gene-Level Coefficients and Hazard Ratios for Two-Gene and Four-Gene Models, related to Figures S2A and S2B

Table S3. Performance comparison of predictive models in EBV-positive GC cohort using 5 × 5 nested cross-validation, related to Figure 6A

Table S4. Pairwise ΔC-index comparisons between predictive models in nested cross-validation and bootstrap validation, related to Figure 6

Table S5. Time-dependent ROC analysis at 24-month horizon using out-of-fold predictions, related to Figure 6B

Table S6. Out-of-fold calibration performance at 24 months, related to Figure 6C

Table S7. Proportional hazards assumption testing for Cox regression models, related to Figure S3A

Table S8. Comparison of time-dependent AUC between data-driven and biologically informed models at 1-, 2-, and 3-year time points, related to Figures S3B and S3C

Table S9. Sensitivity analysis of risk stratification strategies using different grouping approaches, related to Figure S3D

Table S10. Multivariable Cox regression analysis integrating four-gene Immunoscore and tumor stage in EBV-positive GC cohort, related to Figure 7

Table S11. Raw data from transwell migration assays evaluating CD18 blockade, related to Figure 7E

Table S12. Summary of Key Methodological Parameters, related to STAR Methods

Table S13. TRIPOD checklist for the four-gene Immunoscore in EBVaGC, related to STAR Methods
