# Supplementary material for: Immune hub genes and a proof-of-concept prognostic signature in EBV-associated gastric carcinoma
Source: iScience. 2026 Mar 5;29(4):115243. doi: 10.1016/j.isci.2026.115243 (PMC13049670; doi:10.1016/j.isci.2026.115243)
Supplement: Document S1. Figures S1–S4 [file mmc1.pdf]

iScience, Volume *29*

Supplemental information

Immune hub genes and a proof-of-concept prognostic signature in EBV-associated gastric carcinoma

Rui-zhen Huo, Ri-hong Yang, Ri-hua Zeng, Zi-cen Fang, Bin Li, Yu-hang Pan, Jin-rui Guo, Yan-xian Guan, Chun-kui Shao, Dan-ni Yu, Si-hong Liang, Yi-ting Shao, Yu Du, and Jian-ning Chen

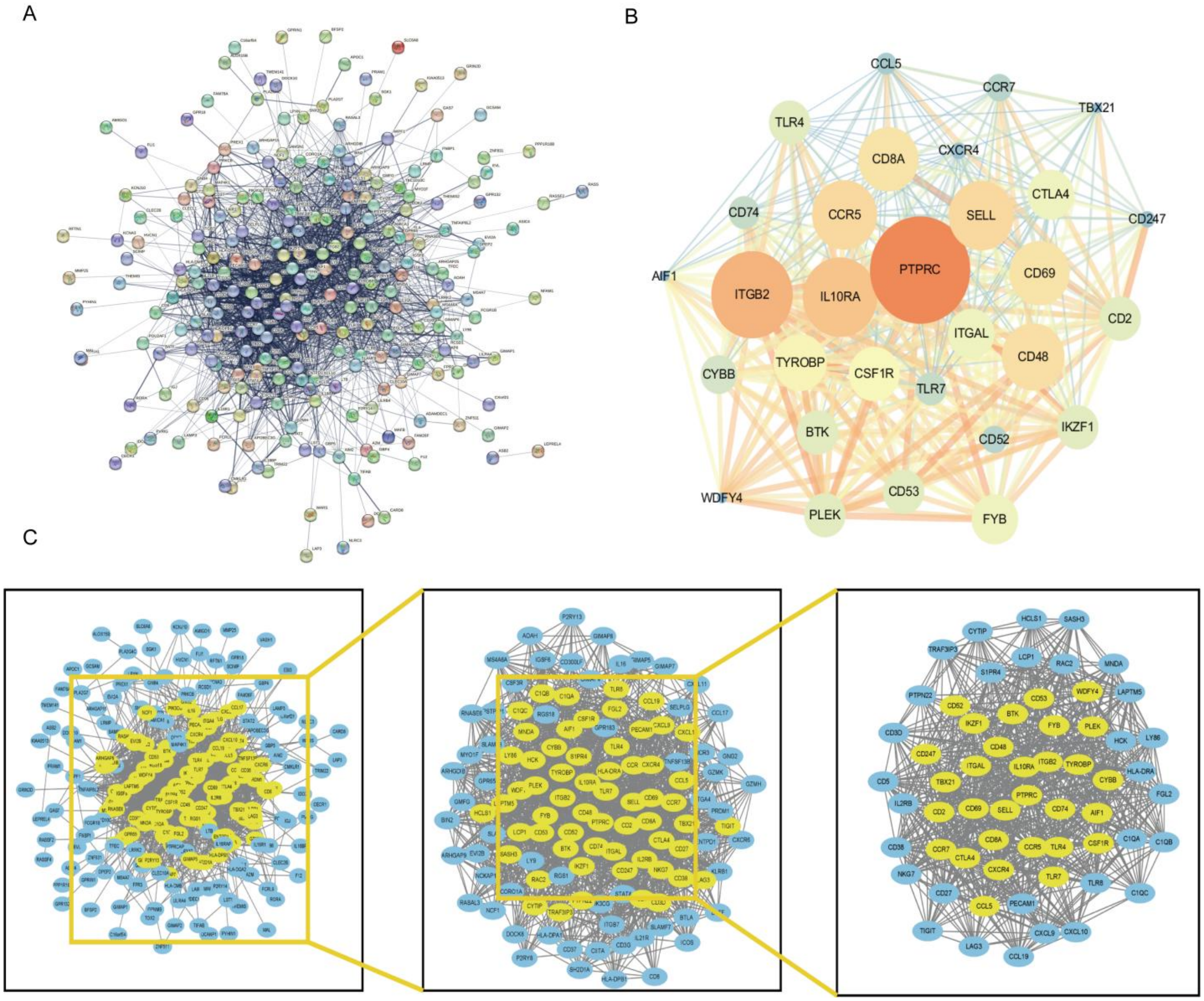

Figure S1. Protein–protein interaction (PPI) network analysis of genes in the brown module. (A) Comprehensive PPI network of brown module genes constructed using the STRING database. Nodes represent proteins, and edges represent predicted functional associations. (B) Three-level zoom-in visualization of key subnetworks with high connectivity. Yellow-highlighted nodes indicate genes with higher degree centrality. (C) Top 30 hub genes identified by the degree algorithm in cytoHubba. Node size and color intensity correspond to the degree value. PTPRC and ITGB2 were the most highly connected hub genes, indicating their central regulatory roles in the EBVaGC immune network.

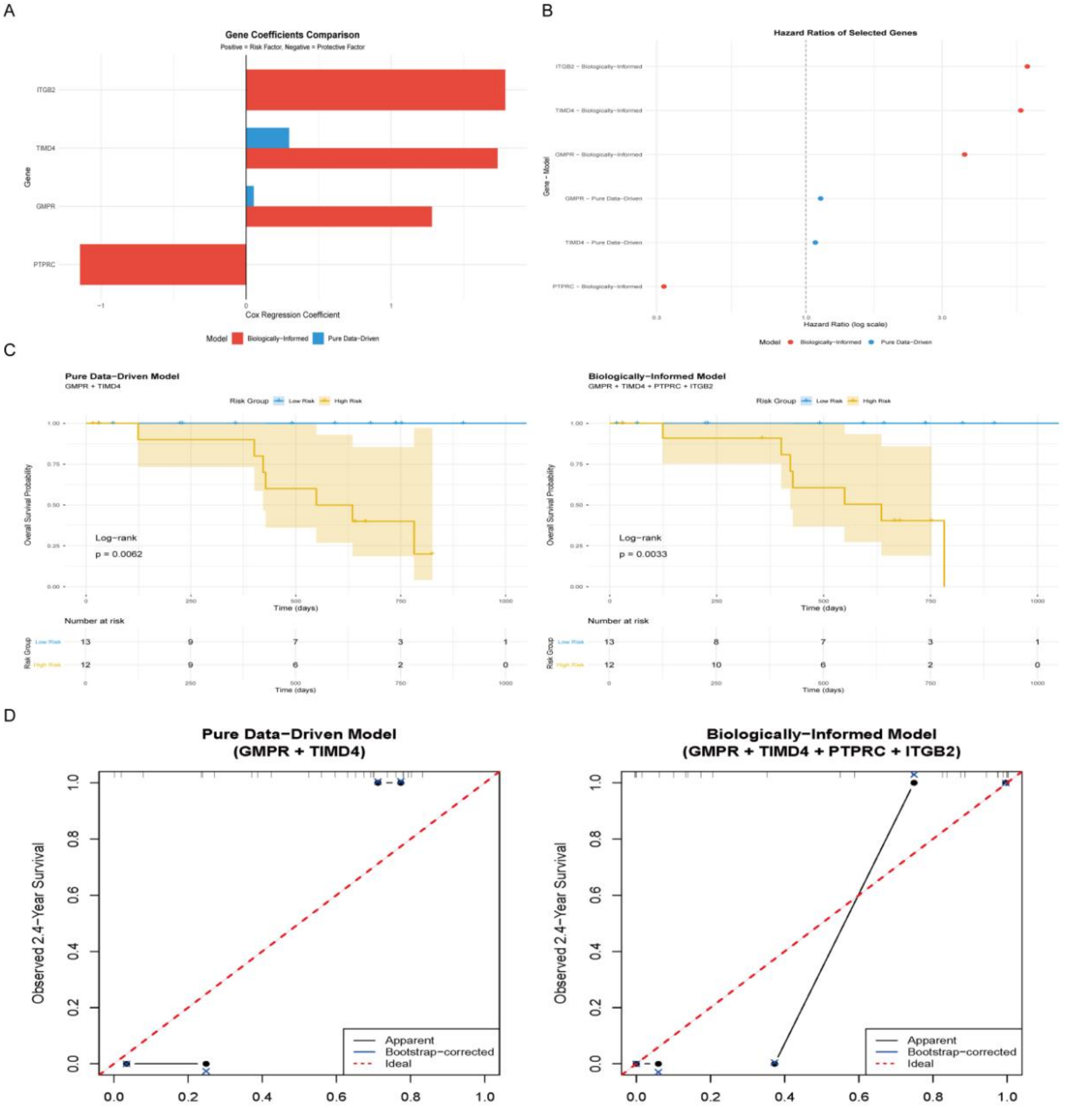

Figure S2. Sensitivity analyses comparing the pure data-driven two-gene model with the biologically-informed four-gene model. (A) Horizontal bar plot showing Cox regression coefficients for each gene in the pure data-driven model (GMPR + TIMD4; blue) and the biologically-informed model (GMPR + TIMD4 + PTPRC + ITGB2; red). Positive coefficients indicate risk factors; negative coefficients indicate protective factors. (B) Forest plot displaying hazard ratios (log scale) of selected genes in both models. Red dots represent genes in the biologically-informed model; blue dots represent genes in the pure data-driven model. Dashed vertical line indicates HR = 1.0. (C) Kaplan-Meier survival curves for overall survival stratified by risk group (low risk vs. high risk) in the pure data-driven model (left; GMPR + TIMD4) and the biologically-informed model (right; GMPR + TIMD4 + PTPRC + ITGB2). Shaded areas indicate 95% confidence intervals. Number at risk tables are shown below each plot. P values are from log-rank tests. (D) Bootstrap-corrected calibration curves for 2.4-year survival prediction in the pure data-driven model (left) and the biologically-informed model (right). Black solid line indicates apparent calibration; blue dashed line indicates bootstrap-corrected calibration; red dashed line indicates ideal calibration. Gray diagonal line represents perfect prediction (observed = predicted).

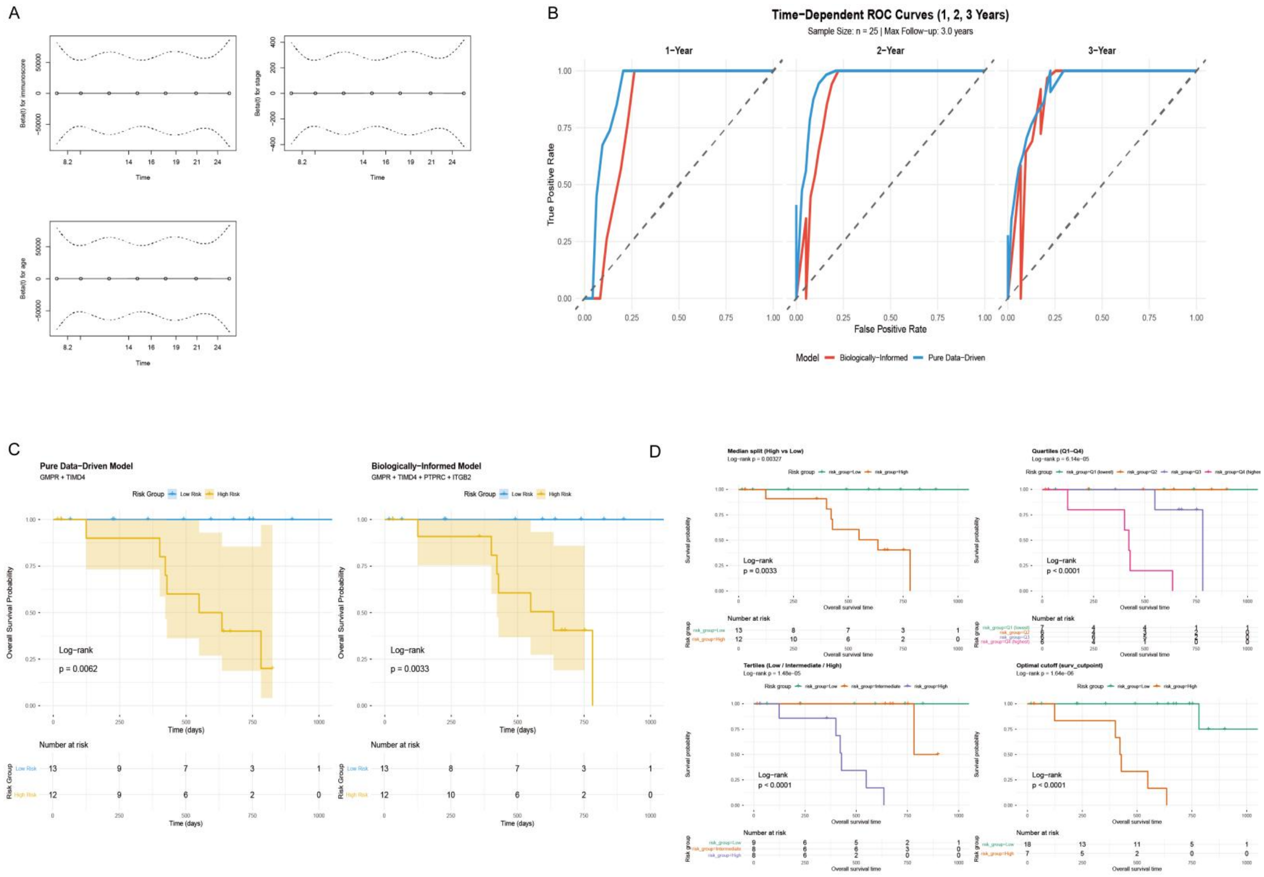

Figure S3. Proportional hazards assumption validation and sensitivity analysis of risk stratification strategies. (A) Schoenfeld residual plots for the immunoscore model showing no significant violation of the proportional hazards assumption across time (global p=0.88). (B) Comparison of 1, 2, 3year ROC curves between biologically-informed and pure data-driven models. (C) Kaplan-Meier survival curves comparing pure data-driven versus biologically-informed models, both demonstrating significant risk stratification (log-rank p=0.0062 and p=0.0033, respectively). (D) Robustness assessment using four prespecified stratification strategies: median split (High vs. Low, p=0.0033), tertiles (Low/Intermediate/High, $p=1.48\times10^{-5}$), quartiles (Q1-Q4, $p=6.14\times10^{-5}$), and optimal cutpoint (low vs. high, $p=1.64\times10^{-6}$), confirming consistent survival separation across all categorization approaches.

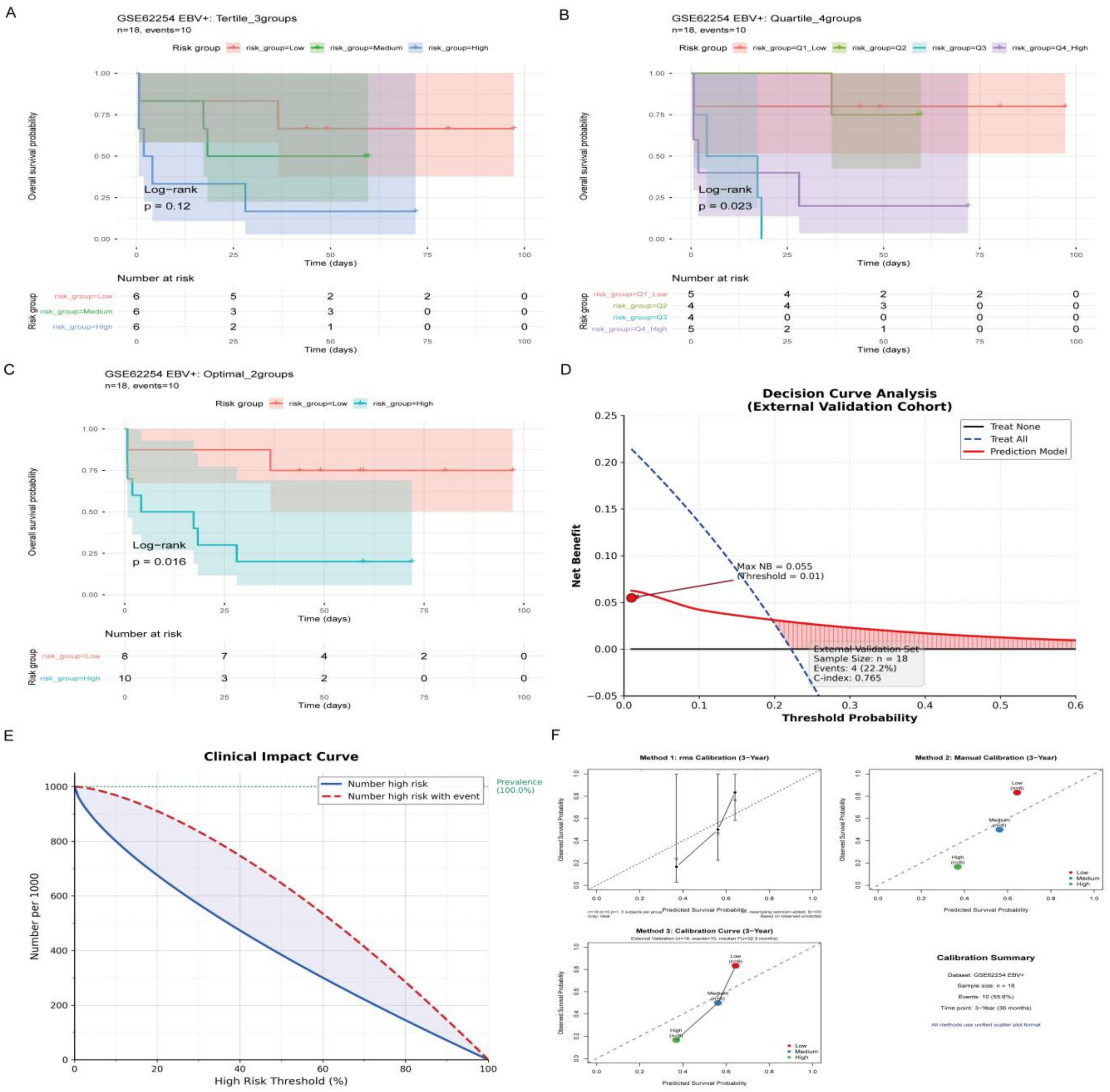

Figure S4. External Validation of the Four-Gene Immunoscore in the GSE62254 EBV-Positive GC Cohort. (A-C) Kaplan-Meier survival curves demonstrating prognostic stratification across multiple grouping strategies: (A) Tertile-based 3-group stratification (log-rank p=0.12); (B) Quartile-based 4-group stratification (log-rank p=0.023); (C) Optimal cutpoint-derived 2-group stratification using surv_cutpoint (log-rank p=0.016, HR=5.69, 95% CI: 1.17–27.58). (D) Decision curve analysis at 24-month horizon showing net benefit of the prediction model (red line) compared to treat-all (black dashed line) and treat-none (grey line) strategies, with maximum net benefit of 0.055 at threshold probability of 0.01. (E) Clinical impact curve illustrating the number of patients classified as high-risk (blue line) and the number of high-risk patients with observed events (red dashed line) across different threshold probabilities. (F) Calibration plots comparing predicted versus observed 3-year survival probabilities in the external validation cohort (GSE62254 EBV+, n=18, events=10). Patients were stratified into tertile-based risk groups. The diagonal line indicates perfect calibration.
